# Supplementary material for: Empirical methods for controlling false positives and estimating confidence in ChIP-Seq peaks
Source: BMC Bioinformatics. 2008 Dec 5;9:523. doi: 10.1186/1471-2105-9-523 (PMC2628906; doi:10.1186/1471-2105-9-523)
Supplement: Additional File 1 — A variety of html documents from the USeq web site detailing the available applications, their best usage, output file type descriptions, command line menus, etc. [file 1471-2105-9-523-S1.zip › USeqUserGuides/JavaDoc.zip!/JavaDoc/trans/roc/Positive.html]

Positive


|  |  |  |
| --- | --- | --- |
| **Overview** \| **Package** \| **Class** \| **Use** \| **Tree** \| **Deprecated** \| **Index** \| **Help** | |  |
| **PREV CLASS**   **NEXT CLASS** | **FRAMES**    **NO FRAMES**     **All Classes** |  |
| SUMMARY: NESTED \| FIELD \| CONSTR \| METHOD | DETAIL: FIELD \| CONSTR \| METHOD |  |


---


## trans.roc Class Positive

```
java.lang.Object
  trans.roc.Positive
```

---

``` public class Positive extends java.lang.Object ```

Container for a positive BAC used by the Sgr scripts.

---

**Constructor Summary**

`Positive(java.lang.String chromosome,
int start,
int stop)`


| **Method Summary** | |
| --- | --- |
| `boolean` | `contains(int position)` |
| `static Positive[]` | `filter(int bpOffSetEnds, Positive[] pos)`             Adds the bpOffSetEnds to the end of each Positive. |
| `java.lang.String` | `getChromosome()` |
| `java.util.ArrayList` | `getScores()` |
| `int` | `getStart()` |
| `int` | `getStop()` |
| `int` | `getTotalNumberWindows()` |
| `boolean` | `isSenseStrand()` |
| `boolean` | `matches(Sgr sgr)` |
| `void` | `setScores(java.util.ArrayList scores)` |
| `void` | `setSenseStrand(boolean senseStrand)` |
| `void` | `setTotalNumberWindows(int totalNumberWindows)` |
| `java.lang.String` | `toString()` |
| `java.lang.String` | `toStringSimple()` |

**Methods inherited from class java.lang.Object**

`equals, getClass, hashCode, notify, notifyAll, wait, wait, wait`

**Constructor Detail**

### Positive

```
public Positive(java.lang.String chromosome,
                int start,
                int stop)
```


**Method Detail**

### matches

```
public boolean matches(Sgr sgr)
```

---


### contains

```
public boolean contains(int position)
```

---


### getScores

```
public java.util.ArrayList getScores()
```

---


### toStringSimple

```
public java.lang.String toStringSimple()
```

---


### filter

```
public static Positive[] filter(int bpOffSetEnds,
                                Positive[] pos)
```

:   Adds the bpOffSetEnds to the end of each Positive.
    If the start < modified end, the Pos is saved.

---


### toString

```
public java.lang.String toString()
```

:   **Overrides:**: `toString` in class `java.lang.Object`

---


### getChromosome

```
public java.lang.String getChromosome()
```

---


### getStart

```
public int getStart()
```

---


### getStop

```
public int getStop()
```

---


### getTotalNumberWindows

```
public int getTotalNumberWindows()
```

---


### setTotalNumberWindows

```
public void setTotalNumberWindows(int totalNumberWindows)
```

---


### setScores

```
public void setScores(java.util.ArrayList scores)
```

---


### isSenseStrand

```
public boolean isSenseStrand()
```

---


### setSenseStrand

```
public void setSenseStrand(boolean senseStrand)
```


---


|  |  |  |
| --- | --- | --- |
| **Overview** \| **Package** \| **Class** \| **Use** \| **Tree** \| **Deprecated** \| **Index** \| **Help** | |  |
| **PREV CLASS**   **NEXT CLASS** | **FRAMES**    **NO FRAMES**     **All Classes** |  |
| SUMMARY: NESTED \| FIELD \| CONSTR \| METHOD | DETAIL: FIELD \| CONSTR \| METHOD |  |


---
